# Relationship between land use type and bacterial composition in adjacent streams and riparian zones

**DOI:** 10.1371/journal.pone.0339590

**Published:** 2026-02-09

**Authors:** Javad Sadeghi, Shayenna Nolan, Patricia Voyer, Zeena Biro, Yasmin Ismail, Emma Mineau, Gina Peters, Alyssa Frazao, Emily Browne, Lauren Weller, Catherine M. Febria, Terrence H. Bell, Daniel Heath

**Affiliations:** 1 Great Lakes Institute for Environmental Research, University of Windsor, Windsor, Canada; 2 Department of Physical and Environmental Sciences, University of Toronto, Toronto, Canada; 3 Department of Integrative Biology, University of Windsor, Windsor, ON, Canada; Albany Museum, SOUTH AFRICA

## Abstract

Anthropogenic activities can negatively impact riparian and stream ecosystems, resulting in declines in biodiversity and certain ecosystem functions. Microbiomes in these environments play crucial roles in primary production, nutrient cycling, and maintaining air, soil, and water quality. While previous studies have examined the effects of land use on streams and soil microbiota, few have evaluated the effects of soil microbiota on aquatic ecosystems based on land use in the riparian systems. In this study, we characterized bacterial composition in six small to moderate-sized streams in both urban and agricultural land use areas using 16S rRNA gene amplicon sequencing and related this to measured physicochemical variables in these environments. Bacterial composition was comparable in soil samples collected at 3m and 1m from the river and in edge and sediment samples, but these differed significantly from bacterial composition in adjacent water. Bacterial alpha diversity (Shannon index) in streams was higher when adjacent to agricultural sites than urban sites, but no effect of land use type on bacterial alpha diversity was observed in soil samples. On the other hand, land use and location had significant impacts on bacterial composition in both soil and water samples. Furthermore, in our sampling sites, stream bacterial composition in agricultural sites was significantly influenced by NH_3_ and NO_3_-NO_2_ concentrations. These findings raise the possibility that aquatic bacterial function may be modified/influenced even when adjacent soil microbiomes appear relatively unaffected. Moreover, given the sensitivity of the water microbiota to land use variation, our results suggest that aquatic bacterial composition and diversity can serve as a powerful bioindicator for assessing riparian impacts on ecosystem health.

## Introduction

Biodiversity is essential for maintaining ecosystem function, but is increasingly threatened by anthropogenic activities such as urbanisation, climate change, and pollutant discharge are placing unprecedented pressure on ecosystem stability and multifunctionality [1,2]. Land use conversion, for instance, has lasting legacy effects on soil ecosystems and is a major driver of biodiversity loss [3]. Microbes, particularly those across land-water transitions, play several crucial roles in driving ecosystem functioning, such as regulating carbon and nitrogen cycling across diverse environments, from soil to freshwater ecosystems [4]. While stream microbial communities exhibit strong spatial and seasonal dynamics in forested streams [5], understanding how microbiomes respond to anthropogenic stressors, and their interactions across dynamic interfaces (such as ecotones) is vital for exploring more nuanced/sensitive indicators of ecosystem health and resilience.

As an ecosystem feature, ecotones are dynamic transitions between distinct ecosystems (e.g., land-water, groundwater-surface water) that shape and support biodiversity and ecosystem functions [6]. Riparian zones, located along the banks of rivers and streams, are ecotonal ecosystems that support diverse plant and animal communities shaped by hydrological and environmental gradients [7,8]. Functioning as natural buffers, they play a key role in water quality, erosion control, habitat provision, and nutrient cycling [9]. Within the riparian zone, soil physicochemical properties, often shaped by land use, are key drivers of microbial composition [10–12]. For example, Middleton *et al* [13] demonstrated that riparian microbial communities can reduce nitrogen loads to adjacent aquatic systems. Additionally, Martin *et al* [14] showed that even a limited level of urban land use change can shift sediment bacterial community composition and functional potential in stream ecosystems. However, how soil physicochemical properties affect aquatic microbiomes remains understudied. An ongoing question is the degree to which differences in aquatic microbial communities are driven by upland soil microbiota and the effect of land use on aquatic ecosystems. Stream microbiota inhabit river and creek waters and are integral to ecosystem function [15]. Streams are often incredibly biodiverse ecosystems, and watershed land use changes decrease stream integrity and water quality, ultimately altering microbial diversity [16]. Investigating the impact of land use changes on riparian zones and the effect of these changes on stream microbiota is essential for informing conservation and management efforts.

To assess the relationships of land use on stream and riparian microbiota, we sampled six sites across urban and agricultural areas in Essex County, Ontario. At each site, soil samples were collected at three distances from the stream (3 m, 1 m, and edge), along with paired stream sediment and surface water samples, enabling analysis of land use effect on both soil and stream microbial composition along a riparian gradient. Bacterial communities were analysed using 16S rRNA gene metabarcoding to determine bacterial community structure and diversity. We tested the following hypotheses: (I) land use will influence bacterial diversity and composition in both soil and adjacent streams, (II) riparian soil bacterial diversity will be more strongly influenced by land use than stream communities. Our results highlight how land use can change the water microbiome even though the effect on soil might be subtle. Moreover, our findings will contribute to a better spatial understanding of riparian zone dynamics, including the impacts of land use on stream microbiomes and demonstrate that aquatic microbial composition and diversity are powerful bioindicators of ecosystem health

## Materials & methods

### Field sampling

**Fig 1:**
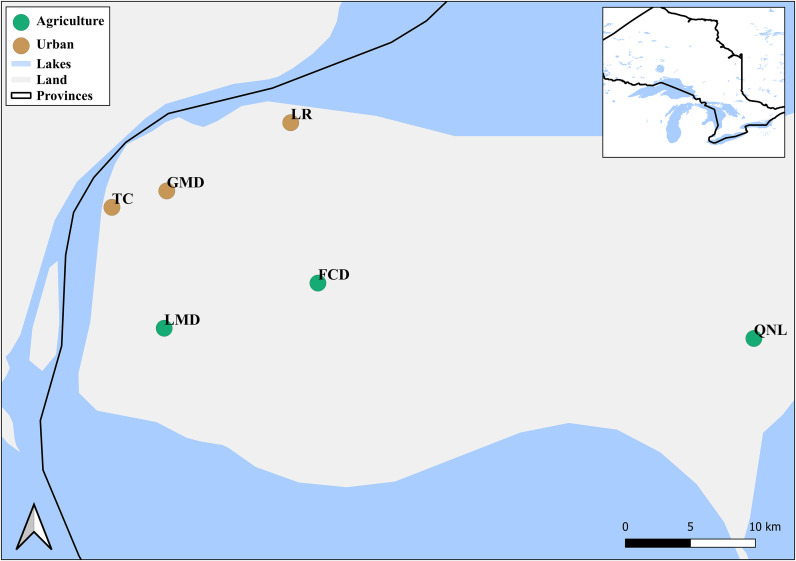
Sampling sites and land use categories in Essex County, Ontario, Canada. Sampling locations are classified as urban (brown circles) or agricultural (green circles). River, lake and basemap were obtained from the Natural Earth database (https://www.naturalearthdata.com/downloads/). Map created using QGIS (v3.40).

Six streams in Windsor-Essex County, Ontario, Canada were sampled from March 19^th^ to 24^th^, 2021 (Table 1; Fig. 1) and assigned to a dominant land use category (urban, agriculture) as described by Nolan *et al* [17]. Briefly, the urban sites were all lined with concrete for a portion of their reaches (for stream) were located within or adjacent to residential areas, whereas the agricultural sites were surrounded by active farmland and characterized by arable land use. Each stream was sampled at two locations ~500-1000m apart. Streams were assigned categorically based on dominant catchment land use. At each sampling location, a 500 mL sample of the top layer of water (0-1m) was taken from the bank, followed by a benthic sediment sample (50 mL). Riparian soil samples (50 mL) were taken from the top 10 cm of soil at the water’s edge, 1m, and 3m into the riparian zone. Sampling was replicated at each sample location, for a total of 2 downstream and 2 upstream sample replicates for each stream (Table 1). Water samples were collected in sterile 500 mL Nalgene™ HDPE bottles rinsed with sample site water before collection. Soil and sediment samples were collected in 50 mL Falcon tubes. Field negative controls (only for water samples) were collected at the beginning and end of sampling to ensure that sampling and transportation did not cause sample contamination. Field negative controls were generated by transferring double-distilled water from one sterile bottle to another and they were stored with the other samples. Samples were stored on ice in a cooler and filtered within two hours of collection.

**Table 1:** Sample Sites: Six streams in Windsor-Essex County with agricultural (AG) or urban (UR) land use were sampled.

Site	Dominant Land Use (%)	Watershed Are (km^2^) [17,18]	Coordinates	Soil								Water		Negative control		Total^b^
**3 Meter**		**1 Meter**		**Edge**		**Sediment**	
**S1** ^**a**^	**S2**	**S1**	**S2**	**S1**	**S2**	**S1**	**S2**	**S1**	**S 2**	**S1**	**S2**
**Grand Marais Drain (GMD)**	UR (83%)	61.1	42.2599, −83.0418	2*	2	2	2	2	2	2	2	2	2	1	1	22
**Little River (LR)**	UR (46%)	64.9	42.3233321, −82.926408	2	2	2	2	2	2	2	2	2	2	1	1	22
**Turkey Creek (TC)**	UR (83%)	61.1	42.244653, −83.092867	2	2	2	2	2	2	2	2	2	2	1	1	22
**Long Marsh Drain (LMD)**	AG (88.8%)	347.8	42.1322, −83.0442	2	2	2	2	2	2	2	2	2	2	1	1	22
**Quinlan Drain (QNL)**	AG (-)	–	42.1227, −82.4953	2	2	2	2	2	2	2	2	2	2	1	1	22
**Fourteenth Concession Drain (FCD)**	AG (88.8%)	347.8	42.1742, −82.9011	2	2	2	2	2	2	2	2	2	2	1	1	22

a. Site (S), b. Number of samples collected at each site.

### Water analysis

To test for environmental effects on water microbiota composition and connectivity, we collected physico-chemical data for each stream. Water quality (pH, dissolved oxygen (DO, mg/L), conductivity (µS/cm), turbidity (FNU), and spatial position (latitude, longitude) were measured using a YSI™ multiparameter probe. Nutrient analyses were performed on samples collected in HDPE bottles following microbial field sampling. Water samples for nitrogen (N) and phosphorus (P) analysis were filtered on site using 0.45µm (VWR®) nylon filters and transported to the laboratory on ice prior to measurement of Nitrate-Nitrite (NO_3_-NO_2_, mg/L), total dissolved phosphorus (TDP, mg/L), and ammonia (NH_3_, mg/L) using a SMARTCHEM® 170 Discrete Analyzer. Water samples for carbon quantification and dissolved organic matter (DOM) analysis were filtered on site using a 0.22µm filter (EZFlow®), transported to the laboratory on ice and stored at 4°C before analysis. Dissolved organic carbon (DOC, mg/L) and total nitrogen (TN, mg/L) were measured using a Shimadzu® TOC-L/TMN-L.

### Dissolved organic matter (DOM) quality and Parallel Factor (PARAFAC) analysis

Water-extractable organic matter (WEOM; considered as a proxy for DOM in soils) was extracted from soil and sediment samples and filtered at 0.22µm, prior to fluorescent DOM characterization [19,20]. DOM spectroscopy was conducted on water and WEOM samples based on established methods [21]. Examining WEOM fluorescence was done by measuring excitation-emission matrices (EEMs) of fluorescence. To identify chemically sounding fluorescent components of organic matter EEM data were sorted using a single multivariate model called parallel factor analysis (PARAFAC) [17,22] using the R package staRdom [23]. Split-half analysis with a Tucker’s congruency correlation value more than 0.98 was used to validate the PARAFAC model [22]. Four components (C1–C4) were identified and validated and complement findings from other local studies [17,20]. Components C1 and C2 are traditionally classified as humic-like components [24,25]. Components C3 and C4 are protein-like components [25,26]. Relative abundances of each component (C1–C4%) were used to assess their effect on soil and water microbiota. Moreover, Based on EEM scans, the following fluorescent spectroscopic indices were calculated: the humification index (hix) which describes the humic content of DOM; the biological index (bix) which indicates autotrophically produced DOM.

### Bacterial DNA extraction and 16S rRNA gene library preparations

eDNA from water (500 mL) and soil samples (250 mg) was extracted by using Qiagen DNeasy^®^ PowerSoil kits according to the manufacturer’s instructions. The V5 (787F-ATTAGATACCCNGGTAG) and V6 (1046R-CGACAGCCATGCANCACCT) variable regions of the 16S rRNA were selected for PCR amplification. [27]. The PCR reactions were conducted in a total volume of 25 μL, consisting of 2.5 μL 10X Taq reaction buffer, 0.5 μL each of 10 μM forward and reverse primers, 0.1 μL of Taq polymerase (5 U/μL), 1.0 μL of 10 μM dNTPs, 3.5 μL of 20 mM MgSO4, and 2.0 μL of extracted eDNA. One lab negative control for each 96 PCR plate (PROGENE^®^) was also included with ultra pure water instead of template DNA. The PCR amplification was conducted after initial denaturation at 95 °C for 3 minutes, followed by 28 cycles of 95 °C for 30 s, 55 °C for 30 s, and 72 °C for 1 min, and a final elongation at 72 °C for 10 min. PCR amplification was verified by visualizing amplicons on an agarose gel. After visualization of PCR products, PCR amplicons were purified using Sera-Mag Magnetic Beads (GE, Healthcare Life Science, UK) and prepared for the second round of short-cycle ligation PCR amplification. All field samples produced bands, whereas negative controls did not (field, DNA extraction, and PCR negative controls). The purified PCR products were used as a template for a second PCR to ligate the adaptor and barcode sequences necessary for sample identification and sequencing. The second short-cycle PCR was conducted in a total volume of 25 μL. The PCR product was visualized on a 2% agarose gel. All samples except the controls had bright bands as a result of 1 μL of the second PCR being combined. Next, the combined samples (132 samples as well as two PCR negative controls (one per plate with only master mix and water and no DNA template) were run on a 1X TE buffer agarose gel for 6 hours, and the band was extracted using QIAquick Gel Extraction Kit. The concentration of purified PCR product mix (library) was measured on an Agilent 2100 Bioanalyzer with a High Sensitivity DNA chip (Agilent Technologies, Mississauga, ON, Canada). The library concentration was then diluted to 60 pmol/μL-1 and sequenced on an Ion PGM™ system using the Ion PGM™ Sequencing 400 bp chemistry and an Ion 318™ Chip (Thermo Fisher Scientific, Burlington, ON, Canada).

### 16S metabarcode sequence data processing

The FASTQ file from the Ion PGM™ system was analyzed using the Quantitative Insights Into Microbial Ecology (QIIME2–2020.11) platform [28]. DADA2 pipeline was used to denoise sequences, dereplicate, filter chimeras, and for Amplicon Sequence Variant (ASV) picking [29]. Taxonomic classification was done through the feature-classifier plugin [30] using the *classify-consensus-blast* command. ASVs related to mitochondrial, chloroplast, and eukaryotic were further removed. Moreover, low abundance taxa (less than 2 ASVs) and ASVs that showed up in only one sample were removed. The 134 samples (120 field samples, 12 field negative controls, 2 PCR negative controls) produced 4,996,367 reads and a total of 6539 ASVs. The PCR negative controls had less than 100 reads. The field negative controls ranged between 0−693 reads. The field samples (120 samples) ranged between 2,393−76,162 reads with a mean frequency of 40,293 reads (Supplementary S1 Fig). Two field samples had less than 3000 reads and the rest had more than 16,000 reads. As a result, the low abundant samples were removed (negative controls as well as two samples from FCD location (FCD soil 1m, FCD water). Shannon’s diversity index (a metric that incorporates both species richness and species evenness) was calculated using QIIME alpha diversity command. β-diversity was calculated using Bray–Curtis dissimilarity index.

### Statistical analyses

#### Bacterial community composition changes.

Soil and water

A bar graph was used to visualize the composition of bacterial communities and their variation among land use and sample locations. Mann-Whitney (MW) U tests (R version 4.4.1) [31] were used to test the effects of land use on microbial community alpha diversity (Shannon diversity). Moreover, Kruskal-Wallis (KW) (R version 4.4.1) tests were also used to test for sampling location (six total: GMD, TC, LR, LMD, FCD, QNL) and origin (3m, 1m, edge, sediment, water) effects on alpha diversity (Shannon diversity). Principal coordinate analysis (PCoA) using the Bray-Curtis dissimilarity matrix was used to visualize the clustering of samples based on sample type (soil vs water). Permutational analyses of variance (PERMANOVA) [32] using *adonis2* in the vegan (v.2.6–4) package in R were used to test for significant differences in beta diversity between soil and water samples. To determine microbiota that are most likely to explain the differences among soil and water samples, linear discriminant analysis (LDA) effect size (LEfSe) [33] was performed based on bacterial taxa at the family-level. LEFSe uses a factorial Kruskal–Wallis and LDA test to detect features with significant differential abundance divergence. Taxa were considered significantly different if their differences had a *P* value < 0.05 and an LDA score (log10) > 3.

Soil

MW tests (R version 4.4.1) were used to test the effects of land use on alpha diversity (Shannon diversity). KW tests were also used for the effect of location (GMD, TC, LR, LMD, FCD, QNL) and sample origin (soil 3m, soil 1m, edge, sediment, and water) on soil alpha diversity. PCoA using the Bray-Curtis dissimilarity matrix was used to visualize the clustering of samples based on location, sample origin, and land use. PERMANOVA using *adonis2* in the vegan (v.2.6–4) package in R was used to test for significant differences in beta diversity among replicate, location, sample origin, and land use. To determine the microbiota that are most likely to explain the differences among soil samples collected from urban and agricultural sites, LEfSe was performed based on taxa at the family-level. Taxa were considered significantly different in abundance if they had a *P* value < 0.05 and an LDA score (log10) > 3.

Stream

The effect of land use and location on stream bacterial alpha diversity was tested using the MW and KW tests, respectively. PCoA using the Bray-Curtis dissimilarity matrix was used to visualize the clustering of samples based on location and land use. PERMANOVA using *adonis2* in the vegan (v.2.6–4) package in R was used to test for significant differences in beta diversity among replicates, location, and land use. To determine the bacterial taxa that are most likely to explain the differences among water sample bacterial communities collected from urban and agricultural sites, LEfSe was performed based on taxa at the family level. Taxa were considered significantly different if their differences had a *P* value < 0.05 and an LDA score (log10) > 3.

### Physico-chemical analysis

#### Soil and water physico-chemical properties.

The effects of physico-chemical parameters of soil (C1percent, C2percent, C3percent, bix, hix) and water (water pH, dissolved oxygen, conductivity, turbidity, NO3-NO2, TDP, NH3, DOC, C1percent, C2percent, C3percent) was tested using the *microeco* R package to calculate Mantel test and Redundancy Analysis (RDA) on soil and water microbiota. Mantel tests were performed (method = “pearson”, permutations = 999, na.rm = TRUE) in R (version 4.3.1) using the vegan (V2.6–4) package to compare the bacterial community Bray–Curtis dissimilarity matrices and geographic distance matrix (*method=”euclidean”*).

## Results

### Riparian soil and stream microbiota diversity differ

**Fig 2:**
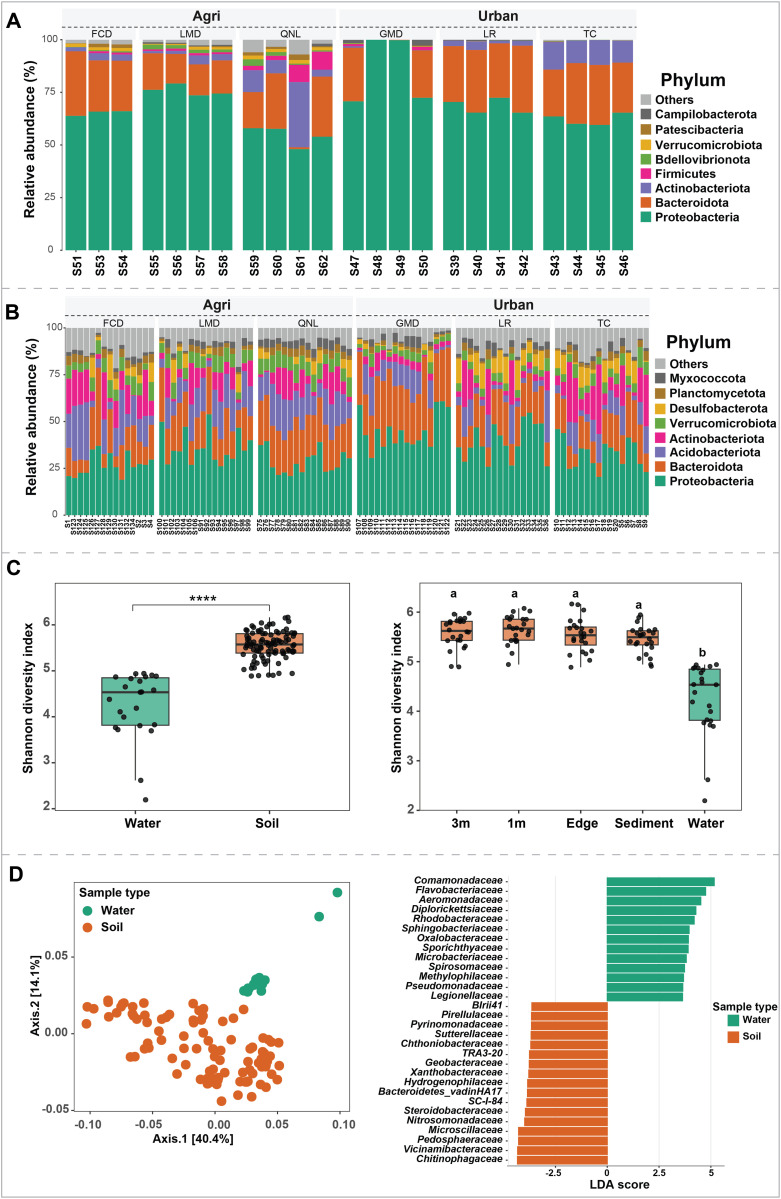
Stream and soil microbiota. Stacked bar plots showing the relative abundance of water (Fig 2A) and soil (Fig 2B) bacterial community composition presented at the phylum level for all 6 sites. Each bar is representative of an individual sample. Panel C: Significant (MW = 2174, P < 0.001) differences in Shannon diversity index between soil and water samples. Panel D (left), PCoA plot showing clustering of samples based on sample type. Panel D (right), bacterial taxa at the family level with significant difference (30 top taxa out of 94 are shown here) between water and soil samples. The significance was assessed using linear discriminant analysis (LDA). Taxa with *P* value < 0.05 an LDA score (log10) > 3 were considered significantly different.

The taxonomic composition of soil and water microbiota was distinct; however, soil and sediment microbiota were most divergent. Proteobacteria, Bacteroidota were the most common phyla across water (Fig 2A) and soil (Fig 2B) samples. At the Family level, the most common bacterial taxa associated with the water were *Comamonadaceae* (Supplementary S1A in S1 Fig). In soil samples, *Chthoniobacteraceae* was the most common bacterial taxon, followed by *Sphingomonadaceae* (Supplementary S1B in S1 Fig). Moreover, soil and water samples collected from urban sites had a higher abundance of *Nitrosomonadaceae* and *Comamonadaceae*, respectively. Samples collected at GMD (both water and soil) showed different microbiota compositions compared to other locations (Supplementary S1A and B in S1 Fig) perhaps due to concrete drain system. Bacterial community diversity was significantly higher for soil samples compared to water (Fig 2C, Shannon: MW = 2174, *P* < 0.001). Moreover, sample origin (3m, 1m, edge, sediment, water) had significant effects on bacterial diversity (Shannon: KW = 57, df = 4, *P* < 0.001) (Fig 2C). Pairwise comparisons showed significant differences between the soil samples (3m, 1m, edge, sediment) and water, but there was no significant differences among soil samples (3m, 1m, edge, and sediment). The PCoA plot based on the Bray-Curtis dissimilarity matrix showed that the stream (water) microbiota clustered separately from the soil microbiota, indicative of different community compositions (Fig 2D). PERMANOVA analyses confirmed the statistical significance of the PCoA clusters (PERMANOVA pseudo-F: 3, *P* value < 0.001). LDA scores showed significant bacterial differences between soil and water samples. At the family level, 94 taxa were found to have significantly different abundances between soil and water. For example, *Comamonadaceae*, *Flavobacteriaceae*, *Aeromonadaceae*, *Diplorickettsiaceae*, and *Rhodobacteraceae* were enriched in water samples while *Chitinophagaceae*, *Vicinamibacteraceae*, *Pedosphaeraceae*, *Microscillaceae*, and *Nitrosomonadaceae* were at high abundance in soil samples (Fig 2D).

### Riparian soil microbiota diversity does not vary with land use

**Fig 3:**
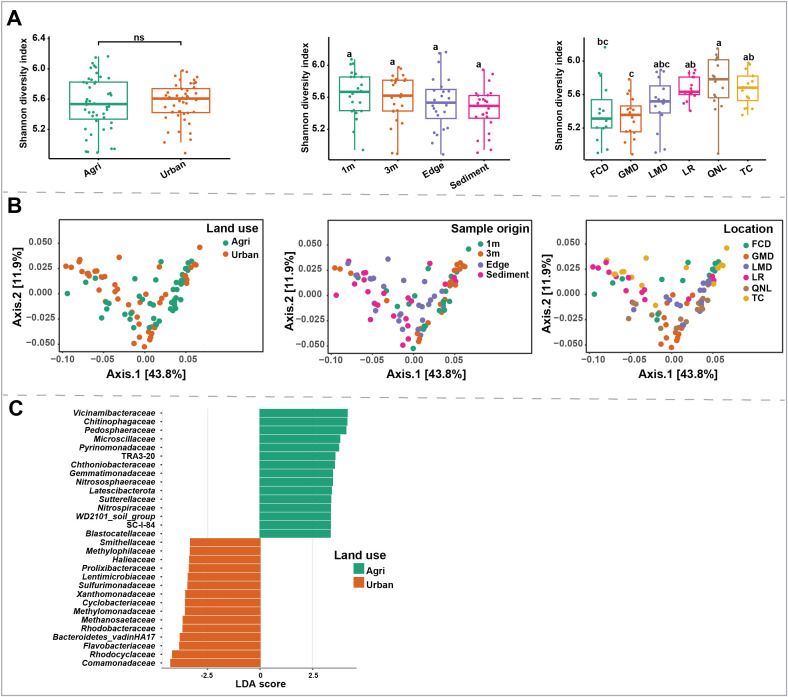
Soil microbiota. Panel A: Shannon diversity index based on land use and sample origin (samples collected at 3m, 1m, edge, and sediment) showed no significant differences. On the other hand, bacterial diversity was different based on where the samples were collected, with QNL showing the highest richness and GMD the lowest (KW statistic: 25.69, *P* < 0.001; results of pairwise comparisons between the locations are presented in Supplementary S1 Table). Panel B: PCoA plot showing clustering of samples based on land use, sample origin, and location. Panel C: Taxa at the genus level with significant differences based on land use that have an LDA score greater than 3.

There was no significant difference in soil microbiota alpha diversity based on land use (Fig 3A, supplementary Fig. S2A in S2 Fig), indicating agricultural sites had similar richness and evenness compared to urban sites. Moreover, sample origin (Fig 3A, supplementary Fig. S2B in S2 Fig) did not show any significant effects, indicating that the soil at 3m, 1m, edge, and sediments had similar alpha diversity. However, locations (Fig 3A, supplementary Fig. S2C in S2 Fig, Supplementary S1 Table) showed significant differences (KW statistic: 25.69, *P* < 0.001) in bacterial alpha diversity. QNL showed the highest diversity, and GMD showed the lowest diversity. Although PCoA plots based on land use, sample origin, and location (Fig 3B) showed considerable overlap, our PERMANOVA analysis showed that land use (PERMANOVA pseudo-F: 2.6, *P* value < 0.001), and location (PERMANOVA pseudo-F: 2.4, value < 0.001) had a significant effect on soil microbiota community variation based on Bray–Curtis dissimilarity (Table 2). On the other hand, replicate (sample replicate) and sample origin did not have a significant effect on soil microbiota composition. LefSe analysis revealed that these differences were mainly driven by changes in 45 taxa at family level (Fig 3C, top 30 taxa are shown). Taxa such as *Vicinamibacteraceae*, *Chitinophagaceae*, *Pedosphaeraceae*, *Microscillaceae* were more abundant in agriculture sites, while *Comamonadaceae*, *Rhodocyclaceae*, *Flavobacteriaceae*, *Bacteroidetes_vadinHA17* were more abundant in urban areas.

**Table 2:** PERMANOVA results for the effects of sample type, land use, sample origin, replicate, and location on microbiota community beta diversity (Bray–Curtis dissimilarity matrix) for both soil and water samples.

Variables	Df	Sum of Sqs	R2	F	Pr(>F)
Sample type (soil vs water)	1	1.16	0.02	3.0216	0.002**
Soil samples					
Land use	1	0.93	0.02	2.61	0.001 ***
Sample origin	3	0.85	0.02	0.78	0.914
Replicate(nested)	3	1.05	0.03	1.04	0.354
Location	5	4.16	0.12	2.46	0.001 ***
Water samples					
Land use	1	1.48	0.21	5.84	0.001 ***
Replicate(nested)	3	0.44	0.06	1.04	0.384
Location	5	4.39	0.64	6.13	0.001 ***

### Stream microbiota diversity is higher in agricultural sites

**Fig 4:**
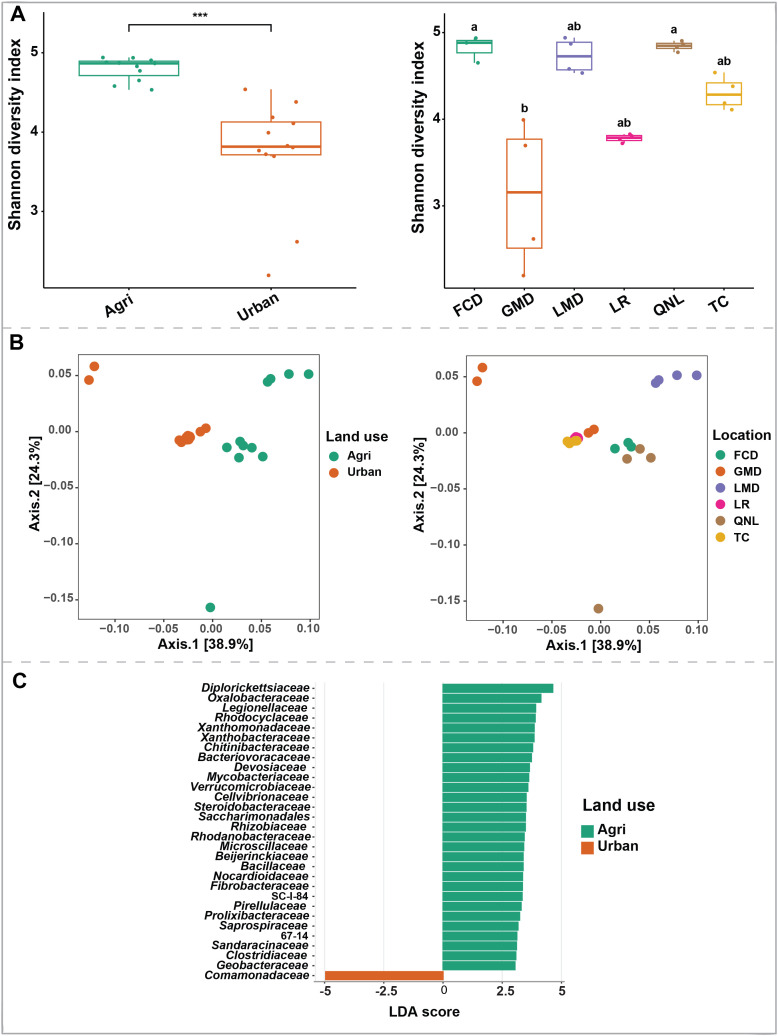
Stream microbiota. Panel A: bacterial alpha diversity (Shannon index) based on land use and location showed significant differences with agricultural sites showed higher diversity compared to urban sites (land use: MW = 1.00, P < 0.001, locations: KW statistic: 18.59, P < 0.01). Panel B: PCoA plot showing clustering of samples based on sample land use and location. Panel C: Taxa at the genus level with significant differences based on land use that have an LDA score larger than 3.

Unlike the soil microbiota, there was a significant effect of land use on the stream microbiota alpha diversity (Fig 4A, supplementary Fig. S3A in S3 Fig) with agricultural sites having higher diversity compared to urban sites (MW = 1.00, *P* < 0.001). Locations (Fig 4A, supplementary Fig. S3B in S3 Fig) also showed significant differences in bacterial diversity. FCD, LMD, QNL (agricultural sites) showed the highest diversity, while GMD was the least diverse site (KW statistic: 18.59, *P* < 0.01, Supplementary S2 Table). PCoA plots showed clear clustering based on land use, and location (Fig 4B). Moreover, our PERMANOVA analysis based on Bray–Curtis dissimilarity showed that land use (PERMANOVA pseudo-F: 5.8, *P* value < 0.001), and location (PERMANOVA pseudo-F: 6.13, value < 0.001) had significant effects on stream microbiota (Table 2). Replicate (sample replicate) did not have a significant effect on stream microbiota. Then we further confirmed the differentially abundant taxa by LEfSe, which identified 28 discriminative features (LDA score ≥ 2) with relative abundance varied significantly between the urban and agricultural sites. For example, agricultural sites had enriched microbial taxa such as *Diplorickettsiaceae*, *Oxalobacteraceae*, *Legionellaceae*, and *Rhodocyclaceae,* while only *Comamonadaceae* was enriched in urban sites (Fig 4C)*.*

### Soil and Water Physico-Chemical Properties

**Fig 5:**
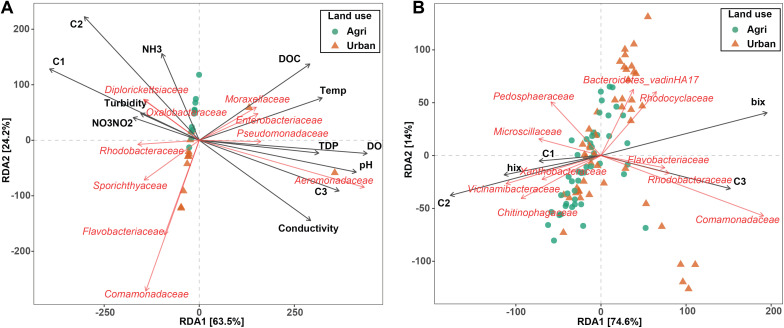
Redundancy analysis (RDA) of ASVs of water (A) and soil (B) samples. RDA plots are displayed based on Bray-Curtis dissimilarity matrices showing the separation of samples by environmental parameters. C4 was removed because of linearly dependent (collinear) with other variables.

RDA was applied to determine the effects of water (Fig 5A) and soil (Fig 5B) physico-chemical properties on the structure of soil and water bacterial communities. For water samples, turbidity, NH_3_, and NO_3_-NO_2_ separated samples based on their land use (Fig 5A), showing higher effects on samples isolated from agricultural sites. Moreover, members of *Diplorickettsiaceae*, *Oxalobacteraceae*, and *Rhodocyclaceae* also showed an association with these samples. Our Mantel test also showed a significant association between the environmental parameters and water microbiota (Mantel statistic r: 0.1679, significance: 0.047). For soil samples C1, C2, and hix had more effects on soil from agricultural sites, while bix, and C3 had more effects on soil from urban sites (Fig 5B). Based on Mantel tests using data from the soil samples, there was no significant association between the environmental parameters and soil microbiota (Mantel statistic r: 0.016, significance: 0.27).

## Discussion

This study sought to characterize the distribution of stream and riparian soil microbiota among two land use types and showed how categorical assignments of land use affects riparian soil microbiota relative to their associated stream microbiota. Microbiota diversity varied significantly across geographic regions in Essex County, Ontario, highlighting the impact of anthropogenic land uses on headwater stream microbial communities and their adjacent riparian zones. Likewise, stream microbiota exhibited regional differences and was strongly correlated with environmental factors such as NH_3_ and NO_3_-NO_2_ concentrations, which distinctly separated agricultural sites from urban areas. More importantly our results reveal that stream microbiota are more variable than soil. These findings align with previous studies, reinforcing dynamics of flowing water systems and the coupled roles of land use in shaping microbial community composition in streams [34–36].

### Soil vs stream microbiota

The dominant bacterial phyla across all sampling sites were consistent with typical 16S rRNA-based analyses of soil and freshwater microbial diversity [27,37–39]. These include Proteobacteria (Pseudomonadota), Bacteroidota, Actinobacteriota, and Acidobacteriota, all of which play essential roles in nutrient cycling (carbon and nitrogen) and overall ecosystem maintenance [40,41]. While their presence was expected, it also indicates that both soil and stream microbial communities are influenced by human activities [42–44]. In this study, microbial alpha diversity was higher in soil than in water, a pattern also observed in previous research [45,46]. Additionally, significant differences were observed between stream microbiota community composition and those from soil, including sediment and edge samples. This suggests that soil microbial communities are more consistent across space, whereas water bacterial communities exhibit greater divergence.

### Land use effect on soil microbiota

Our soil samples did not exhibit a significant land use effect on alpha diversity (Shannon index) when comparing agricultural and urban sites. This finding aligns with Marting *et al*. [14], who also reported that sediment microbiota richness remained unaffected by increasing anthropogenic land use, despite distinct community compositions across sites. However, when assessing beta diversity, we found significant differences based on both land use and location (Table 2). Similarly, Liao *et al* [47] demonstrated that microbiota beta-diversity was strongly influenced by habitat and watershed land use. At the family level, agricultural sites showed a higher relative abundance of *Vicinamibacteraceae*, *Chitinophagaceae*, *Pedosphaeraceae*, *Microscillaceae*. This is consistent with Borsodi *et al.* [48], who reported elevated levels of *Vicinamibacteraceae* in arable fields. Members of *Vicinamibacteraceae* are functionally versatile, with broad substrate preferences, making them essential for maintaining soil ecosystem functions [49]. Members of *Vicinamibacteraceae* contribute to carbohydrate and complex organic matter degradation by secreting various enzymes to obtain energy and carbon sources [50]. In contrast, urban sites exhibited greater abundances of *Comamonadaceae*, *Rhodocyclaceae*, *Flavobacteriaceae*, and *Bacteroidetes_vadinHA17*. *Comamonadaceae*, often associated with nutrient-rich environments, are widespread across various habitats, including soils, activated sludge, and wastewater. They have also been identified as one of the most urban-tolerant bacterial taxa [51,52]. *Rhodocyclaceae*, known for containing numerous denitrifying species, play a crucial role in nitrogen cycling by reducing NO_3_^−^-N to nitrogen (N_2_), and are commonly found in wastewater-impacted environments [53]. Similarly, Zhang *et al*. [54] reported that *Bacteroidetes_vadinHA17* was enriched in industrially polluted environments. These findings suggest that urban sites may provide favorable conditions for denitrifiers (*Comamonadaceae*, *Rhodocyclaceae*, *Flavobacteriaceae*, and *Bacteroidetes_vadinHA17*), potentially making them more dominant than nitrifiers in these environments. However, our Mantel correlation analyses of soil physicochemical conditions with the microbial taxa did not identify a significant association between the environmental parameters and soil microbiota. One reason for this might be that we measured limited number of physicochemical parameters for soil samples.

### Land use effect on stream microbiota

Consistent with previous studies [47,55], urban streams exhibited lower alpha diversity than agricultural streams (Fig 4A), regardless of location (Fig 4A), likely due to increased pollution and habitat degradation [56]. Liao *et al*. [47] also reported that while sediment microbial communities generally displayed higher mean alpha-diversity than water communities, this pattern did not hold for forested sites, where both water and sediment communities had comparable diversity indices. This suggests that water microbiota can be more vulnerable to disturbances (e.g., anthropogenic activities) than soil communities. Our findings support this trend, showing that water microbiota are more sensitive to land use than soil microbiota (Water: R^2^ = 0.21, F = 5.84, Soil: R^2^ = 0.02, F = 2.61). Additionally, several abundant and widespread taxa found in both urban and agricultural streams are commonly associated with high-nutrient, low-oxygen environments indicating eutrophication. Taxa such as *Diplorickettsiaceae*, *Oxalobacteraceae*, and *Rhodocyclaceae* were enriched in agricultural sites. Members of *Diplorickettsiaceae*, belonging to the phylum of Pseudomonadota, have been identified in the soil microbial community and are responsible for the detoxification of metalloids [57,58], suggesting a potential source from agricultural soils inflowing stream water. *Rhodocyclaceae* includes genera like *Dechloromonas* and *Azospira*, known for their role in nitrogen cycling (e.g., denitrification) [59]. This may explain its prevalence in agricultural streams with high nitrate concentrations. The only family that was enriched in urban streams was *Comamonadaceae*. Members of the order *Burkholderiales* (families *Alcaligenaceae* and *Comamonadaceae*) are abundant in urban streams and correlate strongly with several anthropogenic nutrients. *Comamonadaceae* are frequently associated with high-nutrient conditions [52,60] and have been linked to urban streams [51,61] where they exhibit the highest number of urban-tolerant taxa [51,62]. Our Mantel test revealed a significant association between the environmental factors and water microbiota (Mantel statistic r: 0.1679, significance: 0.047). Given the sensitivity of water microbiota to land use variation, these results suggest that land use conversion exerts a greater influence on aquatic microbial communities than on soil communities.

## Conclusion

Like larger organisms, microbes respond to environmental disturbances and are significantly shaped by land use in both soil and water. As a result, their distribution can serve as an indicator of stream and soil conditions. In this study, differences in microbial abundance between urban and agricultural sites were more pronounced in stream samples than in soil, with aquatic microbiota in urban sites exhibiting reduced alpha diversity and distinct community compositions compared to agricultural streams. The enrichment of denitrifying and pollution-associated taxa in urban areas, alongside the stronger correlation between environmental variables and water microbiota, underscores the heightened sensitivity of aquatic systems to anthropogenic disturbance. This stresses the importance of monitoring microbial assemblages in streams as early indicators of anthropogenic impact and highlights the ecological vulnerability of aquatic systems to land use change.

## Supporting information

S1 Fig Water and soil microbiota. Stacked bar plots showing the relative abundance of water (A) and soil (B) bacterial community composition presented at the family level for all 6 sites. Each bar is representative of an individual sample. S and R in panel A indicate sample ID (S) and replicate (R) number. In Panel B, samples are collected at 3 meters (m) away from the river, 1m, edge (E), and sediment (S).
(PNG)

S2 Fig Soil microbiota. Shannon diversity index based on land use (panel A) and sample origin (samples collected at 3m, 1m, edge, and sediment) (panel B) showed no significant differences. On the other hand, bacterial diversity was different based on where the samples were collected (panel C), with QNL showing the highest richness and GMD and FCD the lowest.
(PNG)

S3 Fig Stream microbiota. Bacterial alpha diversity (Shannon index) based on land use (panel A) and location (panel B) showed significant differences with agricultural sites showed higher diversity compared to urban sites.
(PNG)

S1 Table Differences in observed alpha diversity differences in soil samples between locations. Reported *P*-values after Dunn test (Kruskal-Wallis statistic, KW = 25.69, *P* value < 0.001).
(DOCX)

S2 Table Differences in observed alpha diversity differences in water samples between locations. Reported *P*-values after Dunn test (Kruskal-Wallis statistic, KW = 18.59, *P* value < 0.01).
(DOCX)
