# Supplementary figures and images for: Relationship between land use type and bacterial composition in adjacent streams and riparian zones
Source: PLoS One. 2026 Feb 9;21(2):e0339590. doi: 10.1371/journal.pone.0339590 (PMC12885259; doi:10.1371/journal.pone.0339590)

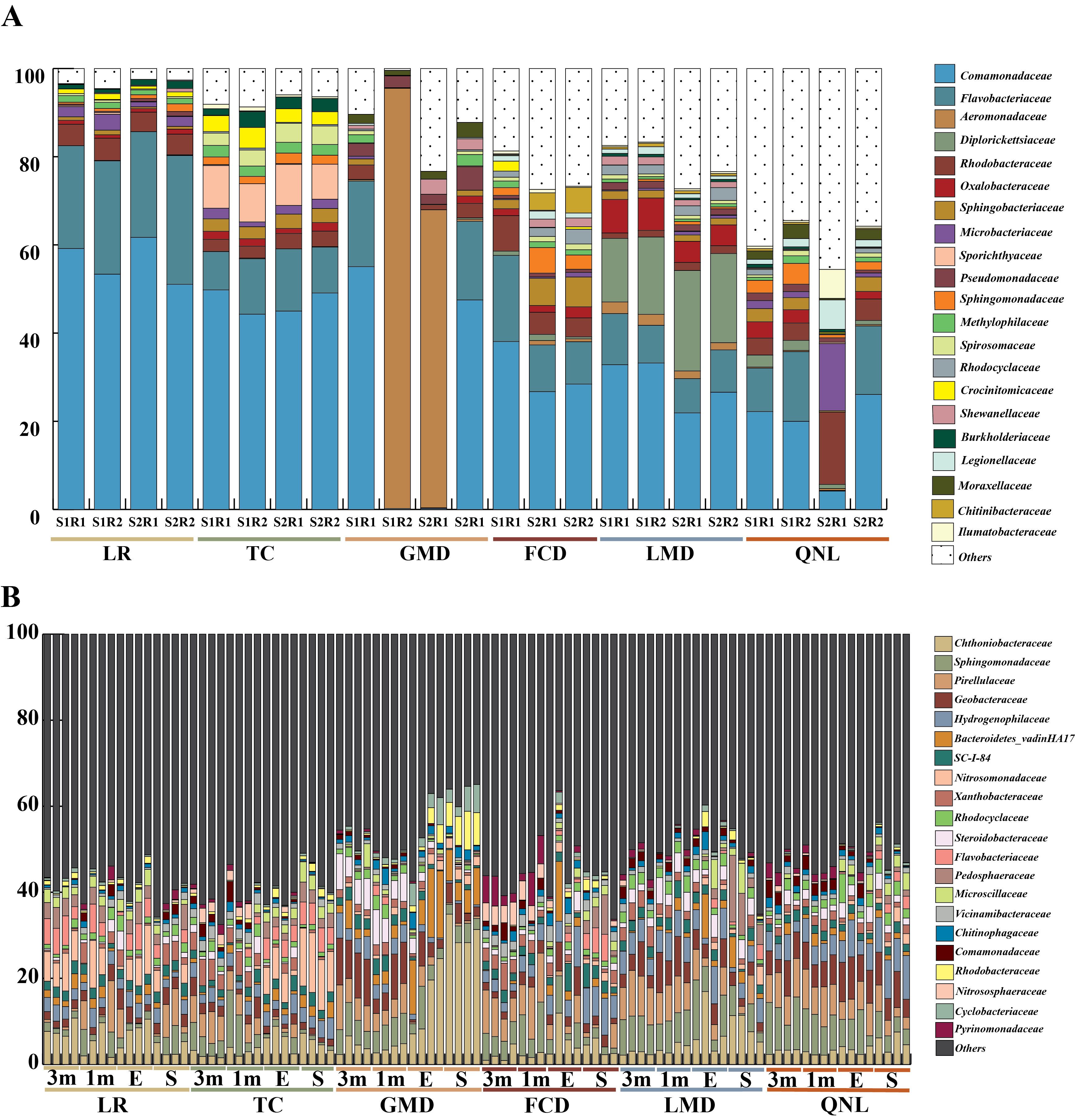

Supplement: S1 Fig — Stacked bar plots showing the relative abundance of water (A) and soil (B) bacterial community composition presented at the family level for all 6 sites. Each bar is representative of an individual sample. S and R in panel A indicate sample ID (S) and replicate (R) number. In Panel B, samples are collected at 3 meters (m) away from the river, 1m, edge (E), and sediment (S). (PNG) [file pone.0339590.s001.png]

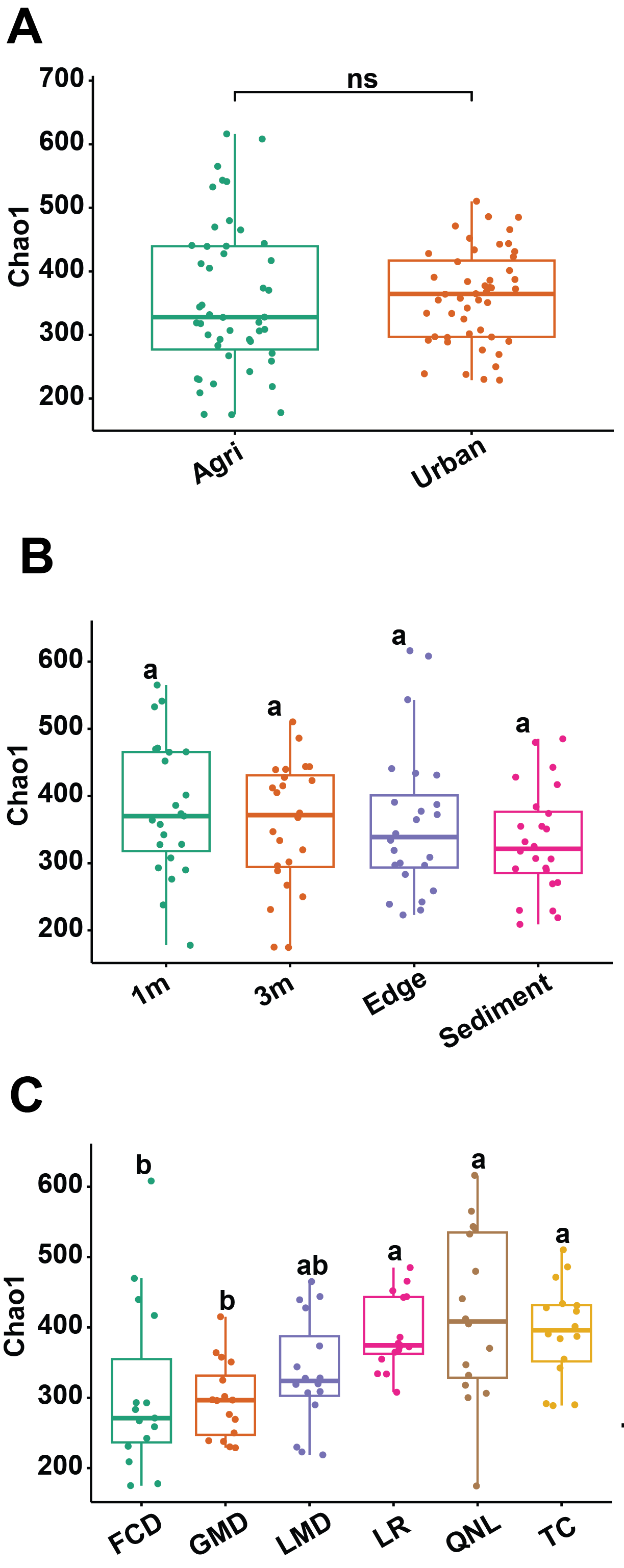

Supplement: S2 Fig — Shannon diversity index based on land use (panel A) and sample origin (samples collected at 3m, 1m, edge, and sediment) (panel B) showed no significant differences. On the other hand, bacterial diversity was different based on where the samples were collected (panel C), with QNL showing the highest richness and GMD and FCD the lowest. (PNG) [file pone.0339590.s002.png]

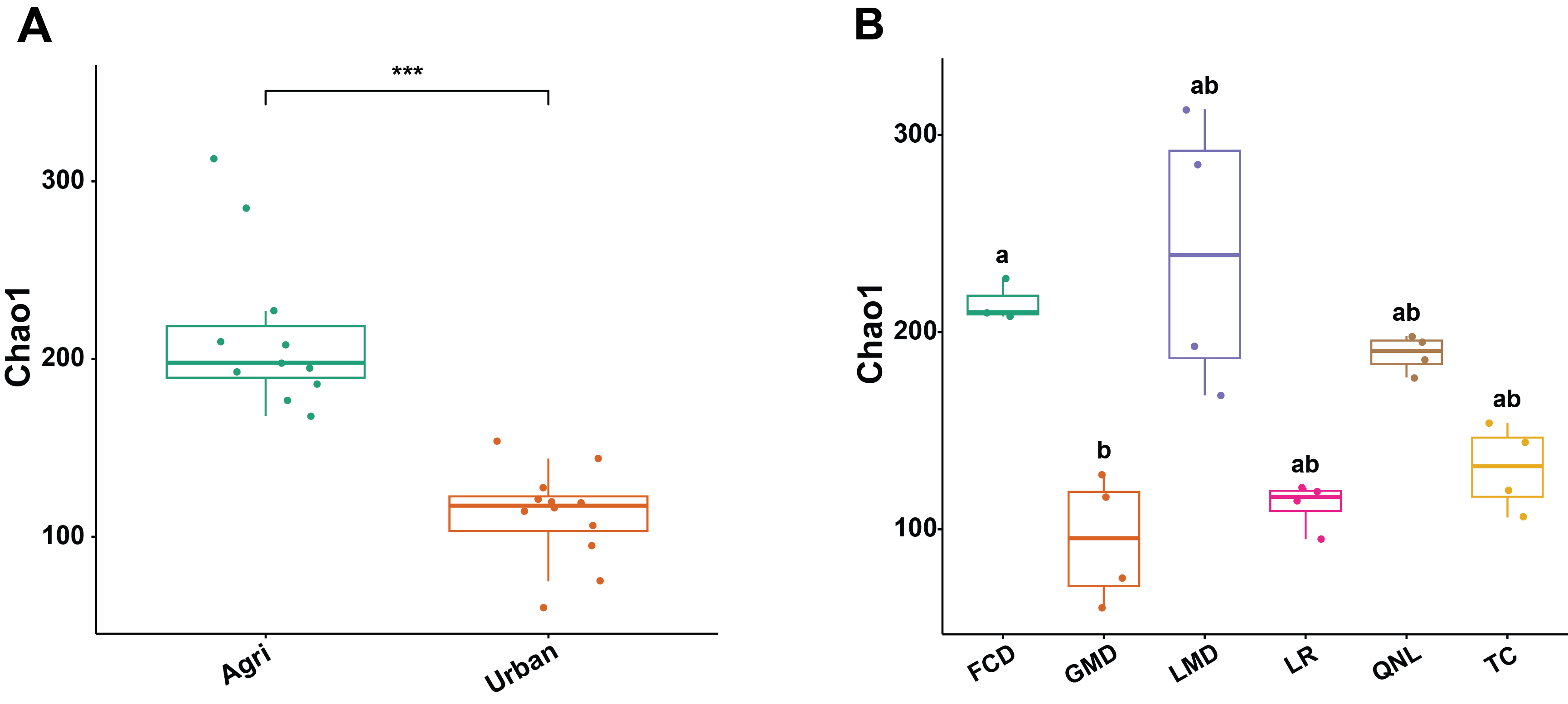

Supplement: S3 Fig — Bacterial alpha diversity (Shannon index) based on land use (panel A) and location (panel B) showed significant differences with agricultural sites showed higher diversity compared to urban sites. (PNG) [file pone.0339590.s003.png]
