# Supplementary material for: Relationship between land use type and bacterial composition in adjacent streams and riparian zones
Source: PLoS One. 2026 Feb 9;21(2):e0339590. doi: 10.1371/journal.pone.0339590 (PMC12885259; doi:10.1371/journal.pone.0339590)
Supplement: S1 Table — Reported P-values after Dunn test (Kruskal-Wallis statistic, KW = 25.69, P value < 0.001). (DOCX) [file pone.0339590.s004.docx]

Supplementary Table S1. Differences in observed alpha diversity differences in soil samples between locations. Reported *P*-values after Dunn test (Kruskal-Wallis statistic, KW = 25.69, *P* value < 0.001).

| Dunn's multiple comparisons test | Mean rank diff. | Significant? | Summary | Adjusted P Value |
| --- | --- | --- | --- | --- |
| FCD vs. GMD | 5.800 | No | ns | >0.9999 |
| FCD vs. LMD | -10.83 | No | ns | >0.9999 |
| FCD vs. LR | -26.33 | No | ns | 0.1183 |
| FCD vs. QNL | -32.08 | Yes | * | 0.0181 |
| FCD vs. TC | -26.83 | No | ns | 0.1017 |
| GMD vs. LMD | -16.63 | No | ns | >0.9999 |
| GMD vs. LR | -32.13 | Yes | * | 0.0147 |
| GMD vs. QNL | -37.88 | Yes | ** | 0.0015 |
| GMD vs. TC | -32.63 | Yes | * | 0.0122 |
| LMD vs. LR | -15.50 | No | ns | >0.9999 |
| LMD vs. QNL | -21.25 | No | ns | 0.4386 |
| LMD vs. TC | -16.00 | No | ns | >0.9999 |
| LR vs. QNL | -5.750 | No | ns | >0.9999 |
| LR vs. TC | -0.5000 | No | ns | >0.9999 |
| QNL vs. TC | 5.250 | No | ns | >0.9999 |
