# Supplementary material for: Relationship between land use type and bacterial composition in adjacent streams and riparian zones
Source: PLoS One. 2026 Feb 9;21(2):e0339590. doi: 10.1371/journal.pone.0339590 (PMC12885259; doi:10.1371/journal.pone.0339590)
Supplement: S2 Table — Reported P-values after Dunn test (Kruskal-Wallis statistic, KW = 18.59, P value < 0.01). (DOCX) [file pone.0339590.s005.docx]

Supplementary Table S2. Differences in observed alpha diversity differences in water samples between locations. Reported *P*-values after Dunn test (Kruskal-Wallis statistic, KW = 18.59, *P* value < 0.01).

| Dunn's multiple comparisons test | Mean rank diff. | Significant? | Summary | Adjusted P Value |
| --- | --- | --- | --- | --- |
| FCD vs. GMD | 15.50 | Yes | * | 0.0415 |
| FCD vs. LMD | 2.000 | No | ns | >0.9999 |
| FCD vs. LR | 13.50 | No | ns | 0.1374 |
| FCD vs. QNL | 1.000 | No | ns | >0.9999 |
| FCD vs. TC | 8.250 | No | ns | >0.9999 |
| GMD vs. LMD | -13.50 | No | ns | 0.0732 |
| GMD vs. LR | -2.000 | No | ns | >0.9999 |
| GMD vs. QNL | -14.50 | Yes | * | 0.0375 |
| GMD vs. TC | -7.250 | No | ns | >0.9999 |
| LMD vs. LR | 11.50 | No | ns | 0.2473 |
| LMD vs. QNL | -1.000 | No | ns | >0.9999 |
| LMD vs. TC | 6.250 | No | ns | >0.9999 |
| LR vs. QNL | -12.50 | No | ns | 0.1372 |
| LR vs. TC | -5.250 | No | ns | >0.9999 |
| QNL vs. TC | 7.250 | No | ns | >0.9999 |
